# Commentary: Glucose Self-monitoring in Non-Insulin-Treated Patients With Type 2 Diabetes in Primary Care Settings: A Randomized Trial

**DOI:** 10.3389/fendo.2018.00389

**Published:** 2018-07-12

**Authors:** Jan Brož, Anna Holubová, Martina Vlasáková, Jan Mužík, Marek Brabec, Dario Rahelić

**Affiliations:** ^1^Department of Internal Medicine, Second Medical Faculty of Charles University, Prague, Czechia; ^2^First Faculty of Medicine, Spin-Off Company and Research Results Commercialization Center, Charles University, Prague, Czechia; ^3^Faculty of Biomedical Engineering, Czech Technical University in Prague, Prague, Czechia; ^4^Institute of Computer Science, Czech Academy of Sciences, Prague, Czechia; ^5^Czech Institute of Informatics, Robotics, and Cybernetics, Czech Technical University in Prague, Prague, Czechia; ^6^Department of Endocrinology, Diabetes and Clinical Pharmacology, Dubrava University Hospital, Zagreb, Croatia; ^7^School of Medicine, University of Zagreb, Zagreb, Croatia

**Keywords:** glucose self-monitoring, type 2 diabetes mellitus, metabolc control, HbA1c, hypoglycemia

The articles published by Young et al. (1, 2) have presented the results and protocol of their Monitor Trial Study, comparing three approaches to self-monitoring of blood glucose (SMBG) to the subsequent outcome of their HbA1c metabolic control, by investigating 3 groups of type 2 diabetes mellitus (T2DM) patients treated with non-insulin antidiabetics, i.e., “no SMBG,” “once daily SMBG,” and “once daily SMBG with enhanced patient feedback” groups.

The authors concluded that neither clinically nor statistically significant differences were found after year 1 of the study, thus expressing a skeptical view toward the routine use of SMBG in type 2 diabetes mellitus (T2DM) patients. A comprehensive list of metanalysis, studies, and recommendations (3) presenting inconsistent results and advice as to the use of SMBG in treating T2DM patients were mentioned in the article The Authors also considered the fact that there was only lower grade evidence (B, C, D) (4) supporting SMBG treatment, including our paper (5) recommending specified SMBG use.

The Monitor Trial Study team has collected and analyzed a large amount of data, leading to interesting and profound debate. Nevertheless, we would like to make three comments that may contribute to further, more detailed discussion of the issue.

1. Clinicians are well aware of the dangers resulting from decision-making based on non-significant statistical results observed outside of carefully designed clinical studies. To this end, it is important to know the study's actual ability to find a significant difference, as expressed by the so-called realized power, i.e., the power evaluated at model parameter estimates. The authors mention that high power was considered in the study planning phase. It seems, however, that: (a) the calculation was not based on the same linear mixed effects model employed for the data analysis; (b) it is crucial to present the realized power calculation for the actual (not the planned) variances, including inter-practice random variability, as well as all the covariates applied in the actual statistical analysis.
2. The study randomized patients with HbA1c values below 7% at the same rate as elderly patients, whose HbA1c goals are often set at higher values. It may be thus speculated that these patients might not have had real interest in improving their HbA1c. This lack of motivation could have led to unchanged HbA1c values or even to their gradual increase, compared to the improved HbA1c results, obtained from motivated patients. Therefore, it would be interesting to include the number of patients with improved HbA1c and the percentage of those with HbA1c < 7% in the analysis.
3. From the clinical experience point of view, we would like to emphasize the statistically significant HbA1c improvement observed at months 3, 6, and 9, and, consequently, to open a discussion about other possible interpretations of the results, namely that SMBG may really prove beneficial only if the patients actually execute the measurements as instructed, since a part of the presented results suggests that the real problem may consist in the poor long-term adherence to the set measurement schedule.

## Author contributions

JB, AH, and MB wrote the commentary. MV, JM, and DR contributed to the design and revised the commentary critically for important intellectual content.

### Conflict of interest statement

The authors declare that the research was conducted in the absence of any commercial or financial relationships that could be construed as a potential conflict of interest.
